# Multi-Output Probabilistic Prediction of Drug Side Effects Using Classical Machine Learning Algorithms

**DOI:** 10.3390/ph19040595

**Published:** 2026-04-08

**Authors:** Diego Quiguango Farias, Juan Sarasti Espejo, Marlene Arce Salcedo, Byron Velasquez Ron

**Affiliations:** 1 Carrera Ciencias de la Salud, Universidad de Las Americas (UDLA), Quito 170516, Ecuador; juan.sarasti@udla.edu.ec (J.S.E.); marlene.arce@udla.edu.ec (M.A.S.); 2 Carrera de Odontología, Universidad de Las Americas (UDLA), Quito 170516, Ecuador; byron.velasquez@udla.edu.ec

**Keywords:** side effects, machine learning, computational pharmacology, predictive models, pharmacovigilance, probabilistic prediction

## Abstract

**Introduction:** Drug side effects are a relevant problem for patient safety and public health, and traditional methods have limitations in capturing complex patterns between clinical and pharmacological variables. **Objective:** To evaluate machine learning models to probabilistically predict multiple side effects associated with drug use. **Materials and methods:** A cross-sectional computational study was carried out with data from 1000 medications that included clinical condition, dosage and duration of treatment. Random Forest, Decision Tree, Support Vector Classifier and KNN were trained and optimized using Grid Search and an 80:20 split for training and testing. Chi-square tests and Principal Component Analysis were applied to explore associations and overlap between categories. **Results:** Significant associations were found between side effects and clinical condition (*p* < 0.05) and the drug administered (*p* < 0.05). The PCA showed a high overlap between categories, which justified a probabilistic approach. Tree-based models showed better performance (accuracy ≈ 0.35). **Conclusions:** Prediction of side effects is a multifactorial and non-deterministic problem; probabilistic machine learning models allow for estimating several plausible adverse events and can support clinical decision-making and pharmacovigilance.

## 1. Introduction

Side effects associated with the use of medicines are one of the main challenges in the field of drug safety and public health, given their direct impact on therapeutic adherence, patient quality of life and the increase in morbidity associated with pharmacological treatments [1]. Despite advances in drug development and pharmacovigilance systems, adverse events continue to be a significant cause of hospitalization and preventable clinical complications [2].

Traditionally, the identification of side effects has been based on controlled clinical trials and passive post-marketing reporting systems. However, these approaches have important limitations, such as low population representativeness, underreporting, and the inability to capture complex patterns between multiple clinical and pharmacological variables [3]. In this context, the use of advanced computational techniques has established itself as a promising alternative to complement classic methods for detecting adverse events [4].

In particular, machine learning has shown high potential for modeling nonlinear relationships between clinical characteristics, dosage, and safety profiles of drugs, allowing the probability of the occurrence of side effects to be estimated in a more precise and personalized way [5]. Several studies have shown that predictive models can outperform traditional statistical methods in the early detection of adverse events, especially when large volumes of heterogeneous data are available [6].

Computational pharmacology thus emerges as an interdisciplinary field that integrates clinical data, pharmacological data, and artificial intelligence algorithms to understand and predict individual response to drugs [7]. Within this framework, the prediction of side effects has become a priority line of research, due to its clinical relevance and its potential to support evidence-based medical decision-making [8].

However, the pharmacological response presents a high interindividual variability, influenced by factors such as age, clinical condition, dose administered and duration of treatment, which makes it difficult to deterministically classify a single side effect per patient [9]. This complexity has driven the development of probabilistic approaches that consider the possibility of multiple concurrent adverse events, rather than single-output models [10].

Additionally, the overlap between categories of side effects represents a relevant methodological challenge for traditional classification systems, since similar configurations of predictor variables can lead to different clinical manifestations [11]. In this sense, tree-based and ensemble-based models have shown superior performance in capturing hierarchical interactions between clinical and pharmacological variables [12].

On the other hand, the integration of probabilistic metrics in the prediction of adverse effects is especially relevant in preventive clinical scenarios, where the main objective is not only to classify an event, but also to identify a small set of potential risks that can be monitored early [13]. This approach is consistent with recent recommendations in pharmacovigilance, which promote the use of predictive models as complementary tools for pharmacological risk management [14].

Consequently, the present research is framed within computational pharmacology and proposes an approach based on classical machine learning models to estimate the probability of occurrence of multiple side effects associated with the use of drugs. This approach responds to the need to develop predictive tools that contemplate the complexity of the pharmacological response, simultaneously incorporating clinical, pharmacological and demographic variables [15,16].

In this way, the study seeks to contribute to the strengthening of clinical decision support systems, providing a probabilistic prediction scheme of adverse effects that allows risk scenarios to be anticipated and patient safety to be improved in the context of pharmacological treatment [17,18].

## 2. Results

### 2.1. Statistical Analysis

The statistical analysis was structured from a global characterization of the data using descriptive statistics and association analysis techniques, with the purpose of evaluating the relevance and degree of relationship between the independent variables in order to avoid information redundancy and the target variable to be predicted. This approach allowed us to identify preliminary patterns and potential dependencies among the variables considered in the study. Likewise, the Chi-square test was used to determine any associations between adverse effects and the patient’s condition or the type of medication prescribed.

In addition, the central axis of the work was oriented towards a predictive approach based on machine learning models, aimed at estimating the probability of the appearance of one or more side effects associated with the consumption of certain drugs. In particular, the prediction strategy was formulated to generate, for each patient or entry setting, a set of the five most likely side effects together with their respective probabilities of occurrence, in order to contribute to the prevention and early detection of potential adverse events.

In addition, in order to explore the possible overlap between the categories of the secondary effect variable, a principal component analysis was carried out to assess the adequacy and robustness of conventional classification metrics, such as accuracy, precision, completeness, and F1-score.

For the optimization of the parameters of the machine learning models, the Grid Search method was used as an iterative training and evaluation procedure, with the aim of maximizing the accuracy value of the models globally. The hyperparameters obtained through this process were later used in the final prediction of side effects in patients.

This procedure allowed for the analysis of the degree of overlap between classes and its potential impact on the discriminative capacity of the models, as well as on the interpretability of traditional metrics, which can be affected when there are shared patterns between multiple response categories.

### 2.2. Findings

Table 1 shows a heterogeneous population in terms of age and treatment regimens, with wide variability in both dosage and duration of treatment. On average, patients are in middle adulthood, while the doses administered are considerably dispersed, reflecting the coexistence of different drug regimens. The duration of treatment is concentrated around an intermediate value, with no marked extremes, suggesting a relatively balanced distribution. Regarding clinical response, most patients achieved moderate to high levels of improvement, indicating a generally favorable therapeutic effect within the analyzed set.

Table 2 shows that the sample had a balanced composition, with a slight predominance of males versus females. Regarding the clinical condition associated with pharmacological treatment, a relatively homogeneous distribution was observed among the categories considered. The most frequent condition was infection, followed by pain, diabetes, hypertension, and depression. These proportions indicate that no single condition dominates the sample markedly, suggesting adequate representativeness of the different clinical scenarios included in the study.

**Figure 1:**
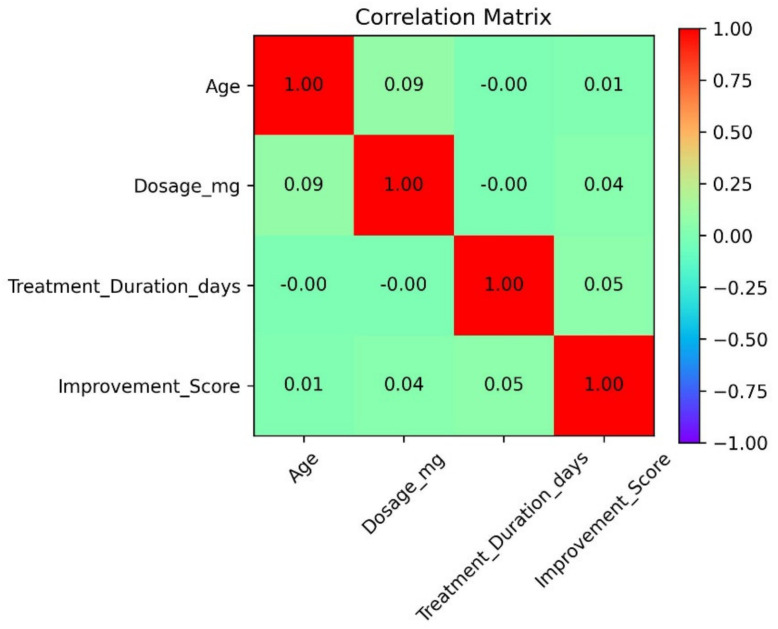
Correlation matrix between numerical predictor variables.

In the present study, one of the relevant methodological aspects corresponds to the analysis of the possible redundancy of information between the predictor variables [19]. In order to identify strong linear relationships that could indicate information duplicity, the correlation matrix between the numerical independent variables was calculated and is presented in Figure 1. The results show the absence of significant correlations between these characteristics, which indicates a low degree of linear dependence between them.

This behavior suggests that there is minimal information redundancy in the set of variables considered, so that each characteristic provides differentiated information to the model without substantially overlapping with other predictor variables. Consequently, independent variables can be incorporated together in machine learning models without a high risk of multicollinearity, thus strengthening the stability of the estimators and the interpretability of the results obtained.

The results in Table 3 show a statistically significant association between side effects and clinical condition (*p* < 0.05) and between side effects and the drug administered (*p* < 0.05).

These findings indicate that the occurrence of side effects is not independent of the patient’s underlying medical condition, but varies significantly depending on the type of pathology treated. Likewise, there is evidence of a significant relationship between the reported side effects and the drug used, suggesting that different active ingredients have differentiated safety profiles.

Overall, these results support the hypothesis that both the clinical condition and the medication are relevant factors in the manifestation of adverse effects, which justifies their inclusion as explanatory variables in the proposed predictive models. The magnitude of the chi-square statistics obtained also suggests a structured and non-random relationship between these variables, reinforcing the probabilistic modelling approach adopted in the study.

**Figure 2:**
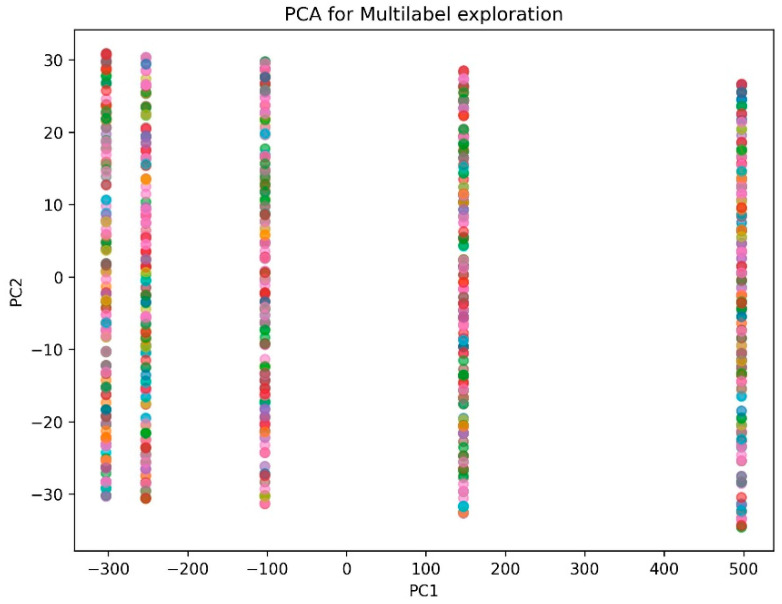
Principal Component Analysis for label overlap analysis.

In order to analyze the overlap between the categories of the side effects variable, a dimensionality reduction procedure was applied using Principal Component Analysis (PCA), the results of which are presented in Figure 2. Through principal component analysis (PCA), a dimensionality reduction process was applied to the numerical predictor variables in order to project the data into two dimensions. Since the problem involves a multidimensional prediction setting, this approach enables the representation of information in a two-dimensional space that would otherwise remain in a high-dimensional feature space. In an ideal scenario, a clear and defined separation between the color groups would allow for the inference of an adequate capacity for discrimination between the different categories of side effects. However, the results show considerable overlap between the groups, without a clear delimitation of regions corresponding to each category.

This lack of clear separation indicates that there are similar configurations of predictor variables that may lead to similar side effects or, in certain cases, to the simultaneous manifestation of multiple adverse effects. This behavior reflects the inherent complexity of the pharmacological response and the interindividual variability in the occurrence of adverse events.

Consequently, these findings support the relevance of an approach based on probabilistic prediction of potential side effects, rather than a strict classification scheme of a single category per patient. This approach is more in line with the nature of the problem addressed, as it allows estimating a reduced set of plausible adverse effects associated with the use of certain drugs, thus strengthening the clinical utility of the model as a support tool for the prevention and early surveillance of adverse events.

**Figure 3:**
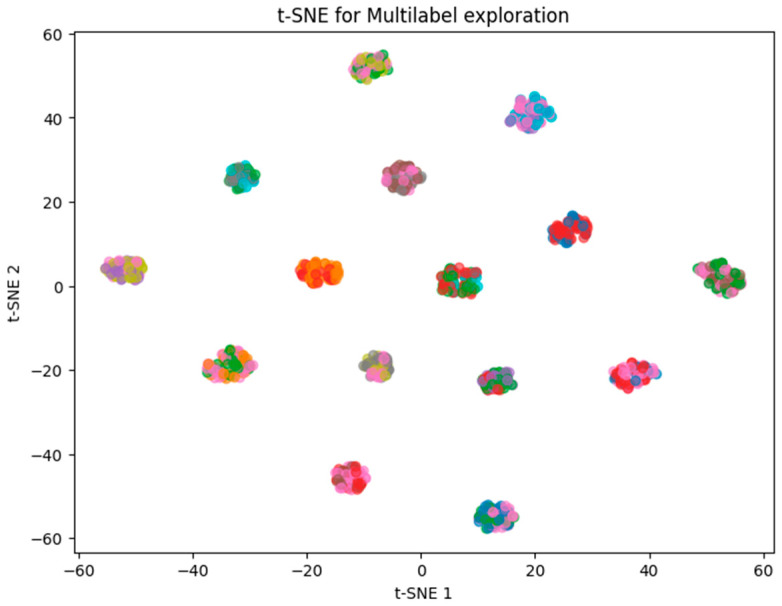
T-SNE for label overlap analysis.

In line with the results derived from principal component analysis (PCA), the grouping obtained through the t-SNE dimensionality reduction technique exhibits a comparable pattern (Figure 3). That is, no distinct separation among side effects is observed; rather, the data form relatively heterogeneous clusters, which reinforces the hypothesis of symptom overlap across records.

Table 4 presents the optimal hyperparameters identified for each of the machine learning models evaluated, along with the value of the metric used during the optimization process. In general terms, it is observed that models based on decision trees (Random Forest and Decision Tree) achieved superior performance compared to distance-based methods (KNN) and nonlinear separation functions (SVC). These results suggest that approaches that exploit hierarchical structures and nonlinear relationships between explanatory variables show a greater ability to capture the complexity inherent in the problem of predicting side effects, even when overlap between categories limits the overall performance of the models.

Table 5 presents the results obtained from the training process using cross-validation. Overall, the results indicate a relatively stable performance across the different models, as evidenced by the standard deviation values associated with each model. Furthermore, the Random Forest model achieved the highest performance metric during the five-fold cross-validation training process, followed closely by the Decision Tree model, which exhibited a relatively similar performance. In contrast, the remaining two models showed considerably lower performance compared with the first two models. The models were trained on a system equipped with an Intel i7 CPU, 12 GB of RAM, without GPU acceleration, and approximately 110 GB of available storage.

Table 6 presents an example of the output generated by the Random Forest model for the top four most probable symptoms, which shows, for each instance evaluated, the side effects with the highest estimated probability, ordered according to their relevance. In general terms, it is observed that the model systematically assigns the highest probability to the main side effect, which indicates a consistent pattern in the hierarchy of predictions. Likewise, there is evidence of a correspondence between the observed side effect and the one with the highest predicted probability, which suggests an adequate internal coherence of the model in the cases illustrated.

In addition, the presentation of multiple side effects per instance allows representing the uncertainty inherent in the predictive process and reflects the clinical possibility of the simultaneous occurrence of different adverse events. This probabilistic approach is especially relevant in the pharmacological field, since it provides a limited set of plausible side effects that can be considered in clinical surveillance tasks and in early prevention strategies, instead of being restricted to a single deterministic prediction [20,21].

As an overview of the performance of the proposed models, the prediction generated by each of the algorithms considered in this study was incorporated, together with the probability associated with the secondary effect estimated as most likely (Table 7). This representation allows direct comparison of the output of the different machine learning approaches and facilitates the clinical interpretation of the results by providing not only a category of expected adverse effect but also a quantitative measure of uncertainty associated with this prediction.

**Figure 4:**
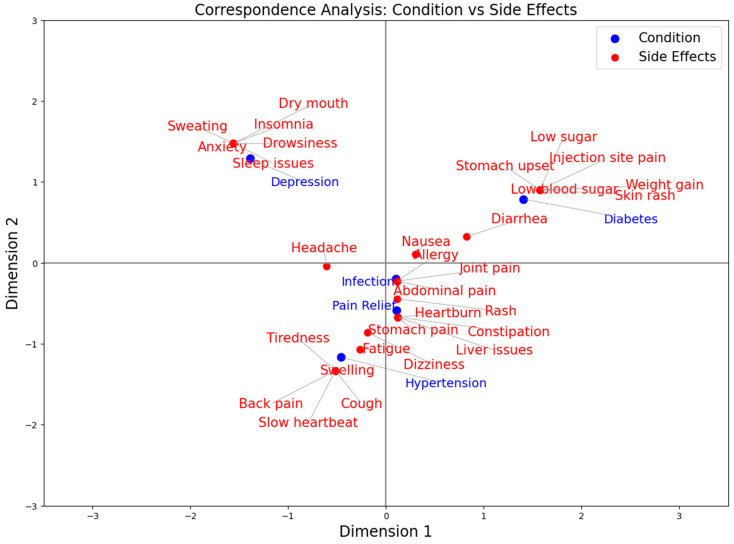
Correspondence analysis for condition vs. side effects.

As shown in Figure 4, patients with depression exhibit greater proximity in the factorial space to effects related to neurological or central nervous system symptoms, particularly anxiety, somnolence, dry mouth, sleep disturbances, and sweating. In the case of diabetes, the most associated side effects mainly correspond to gastrointestinal and metabolic alterations, such as stomach discomfort, diarrhea, and weight gain, in addition to dermatological manifestations. For hypertension, proximity is observed with symptoms related to the cardiovascular and respiratory systems, including bradycardia, cough, swelling, and fatigue. In the case of infections, the most associated effects include abdominal pain and allergic reactions, followed by dermatological manifestations such as rashes. Finally, for pain relief treatments, the most commonly associated side effects mainly correspond to gastrointestinal symptoms, such as abdominal pain and constipation, followed by cutaneous reactions.

Furthermore, Table 8 evaluates the reliability of the predictions of the most probable side effects. A top-k evaluation was performed with k = 1, 3, and 5. This metric assesses whether the true side effect appears as the most probable prediction (top-1), within the three most probable predictions (top-3), or within the five most probable predictions (top-5) for each trained model.

### 2.3. Comparison with Alternative Models

In the previous results, a multi-output prediction approach was implemented using Random Forest, Decision Tree, SVC, and KNN models. However, it is important to note that these are not the only machine learning models that can be applied to this type of problem. Emphasis can be placed on the two best-performing models, namely Random Forest and Decision Tree, which showed favorable classification results by correctly identifying the true side effects within their top-3 predicted outcomes.

Nevertheless, alternative approaches such as XGBoost and ML-KNN can also be considered for multi-output or multi-label prediction tasks. These methods have been widely applied in classification problems and, in many cases, have demonstrated competitive or slightly superior predictive performance compared to traditional machine learning models [22]. However, the computational cost associated with these algorithms may increase as the size of the dataset grows, particularly in the case of gradient boosting methods such as XGBoost [23].

Similarly, ML-KNN has been proposed as an extension of the k-nearest neighbors algorithm specifically designed for multi-label classification problems. This method estimates label probabilities based on the distribution of labels among neighboring instances and has shown competitive performance in several multi-label learning tasks [24]. Nevertheless, in practice, the predictive performance obtained with ML-KNN is often comparable to that achieved by conventional tree-based models such as Decision Trees, especially in datasets of moderate size.

## 3. Discussion

The results obtained in this study confirm the high complexity inherent in the prediction of pharmacological side effects when multiple clinical and pharmacological variables are considered simultaneously. The statistically significant association observed between side effects and both the clinical condition and the medication administered is consistent with previous reports that highlight the non-independent nature of adverse events with respect to the therapeutic profile and pathological context of the patient [25]. This finding reinforces the relevance of modeling such events as multicausal phenomena, rather than assuming them to be isolated responses to a single factor.

The absence of strong correlations between the independent numerical variables suggests that each characteristic provides complementary information to the predictive process, which is consistent with studies that point out the importance of integrating multiple sources of clinical information to improve the explanatory capacity of models [26,27]. This behavior supports the use of machine learning algorithms capable of capturing complex interactions between variables, particularly in scenarios where there is no significant multicollinearity [28].

In relation to model performance, tree-based algorithms (Random Forest and Decision Tree) showed superior performance compared to distance- or margin-based methods, such as KNN and SVC. This pattern has previously been described in pharmacological applications, where ensemble-type models are more robust against heterogeneous data and nonlinear distributions [29]. The ability of these models to identify hierarchical partitions of the feature space is particularly useful in the prediction of adverse effects, where the relationships between variables are usually nonlinear and contextual [30].

However, the observed moderate performance values reflect the intrinsic difficulty of the problem, mainly associated with the overlap between the categories of side effects. This phenomenon has been documented in recent studies that address the prediction of adverse reactions as a multilabel classification problem, in which the same patient may present multiple simultaneous clinical manifestations [31]. The PCA-evidenced overlap supports this interpretation, showing that similar configurations of characteristics can lead to different pharmacological responses [32].

From a methodological perspective, these results justify the use of a probabilistic approach rather than a strict classification scheme. The prediction of the most likely side effects along with their associated probabilities allows capturing the uncertainty inherent in the process and aligns with recent proposals that promote multiple-output models for pharmacological risk management [33,34]. This approach is particularly relevant in preventive clinical settings, where early identification of a small set of plausible adverse effects can facilitate surveillance and therapeutic decision-making [35,36].

Likewise, the interindividual variability observed in the pharmacological response is consistent with evidence that highlights the role of demographic, clinical, and therapeutic factors in the occurrence of adverse events [37]. This heterogeneity limits the usefulness of traditional classification metrics as a final validation criterion, as has been pointed out in studies that warn against biased interpretation of accuracy in multiclass problems with overlap between categories [38,39].

Overall, the results of the present work are in line with recent research that proposes the integration of machine learning models in pharmacovigilance systems as complementary tools to classical signal detection methods [40,41]. The adoption of probabilistic and multilabel strategies is emerging as a solid methodological alternative to address the complexity of side effects by allowing a more realistic representation of clinical scenarios [42,43].

Finally, this study provides empirical evidence that supports the usefulness of predictive models as support instruments for the prevention and early detection of adverse events. In accordance with recent literature, the implementation of systems based on artificial intelligence in pharmacology can contribute to improving patient safety and optimizing therapeutic decision-making, provided that the limitations associated with class overlap and biological variability are considered [44,45].

In this context, it is necessary to specify that this type of tool does not replace pharmacovigilance supervised by a health professional; however, its exploration and improvement constitute support for these professionals by contributing to the improvement of treatments and the prevention of adverse effects in patients.

## 4. Materials and Methods

For the present study, a dataset composed of real pharmaceutical information corresponding to 1000 drugs was used, which includes variables such as the name of the drug, the associated clinical condition, the dosage and the reported side effects, among other relevant characteristics. In order to analyze and predict the potential occurrence of adverse effects, several classic machine learning algorithms were trained, aimed at estimating the probability of occurrence of certain side effects based on a specific configuration of input variables.

The dataset was derived from secondary pharmacovigilance sources that compile adverse drug reaction information from regulatory and drug label repositories, primarily including the U.S. Food and Drug Administration Adverse Event Reporting System (FAERS) and the Side Effect Resource (SIDER). FAERS aggregates spontaneous reports submitted by healthcare professionals, manufacturers, and patients, while SIDER curates adverse effect information extracted from structured drug labels approved by regulatory agencies such as the European Medicines Agency and the FDA. The data were processed through standardization of drug names and adverse effect terms and subsequently aggregated at the drug–symptom level by the data.world corporation, without inclusion of individual patient-level variables (e.g., dosage, treatment duration, or comorbidities). Consequently, the dataset represents reported associations between drugs and adverse effects rather than confirmed causal relationships.

The models considered in this work were: Random Forest (RF), Decision Tree (DT), Support Vector Classifier (SVC) and k-Nearest Neighbors (KNN). In order to maximize the robustness and reliability of the predictions, a systematic hyperparameter optimization process was implemented using the Grid Search method, evaluating multiple combinations of parameters to identify those that would provide the best performance for each model. The split for the train and test datasets was 80:20 for all training processes and hyperparameter optimizations.

The problem addressed is framed within the field of computational pharmacology, where it seeks to predict the potential side effects associated with certain drugs simultaneously, considering the dosage and the previous clinical conditions of the patients. It should be noted that, due to the physiological complexity of the human body and the variability in the biochemical response to drugs, the existence of interindividual differences in the manifestation of adverse effects is expected. Consequently, similar configurations of features may result in different results, including the simultaneous occurrence of multiple side effects.

Within the framework of a single-category deterministic prediction problem, a probabilistic multi-output approach is adopted, in which not only one class is estimated, but a probability distribution is obtained over each of the possible side effects. As a final result, the five most likely side effects are reported.

For this reason, traditional classification metrics, such as accuracy, precision, completeness and F1-score, were not used as the main evaluation criterion but as exploratory tools to analyse the ability of models to maximise the probability of predicting one or more side effects. The predictive strategy adopted was based on probabilistic estimation, so that each trained model produces as output the four side effects with the highest probability of occurrence for a given configuration of input variables.

The information processing and training of the models were carried out using the programming language Python (version 3.12.12) with the help of the Pandas library (version 2.2.2) for the import and manipulation of the dataset, Matplotlib (version 3.10.0) for the construction of the graphs and scikit-learn (version 1.6.1) for the training of the machine learning models through the Google Colaboratory platform. The full source code that allows the reproduction of the results is available in a public GitHub repository, accessible at https://github.com/diegoqfestadistica/Side_Effects.git (accessed on 10 March 2026).

## 5. Conclusions

The present study demonstrates that the prediction of pharmacological side effects is a complex problem, characterized by the overlap between response categories and by the high interindividual variability in the manifestation of adverse events. Statistical analyses showed significant associations between side effects, clinical condition, and medication administered, supporting the inclusion of these variables as relevant predictors. Likewise, the absence of strong correlations between the numerical variables suggests that each characteristic provides complementary information to the predictive process, strengthening the stability of the trained models.

From a methodological perspective, the results obtained indicate that tree-based models perform better than other classical approaches, although the moderate values achieved reflect the limitations inherent to deterministic classification in this context. In this sense, the probabilistic approach adopted, aimed at estimating a reduced set of plausible side effects along with their probabilities of occurrence, is more appropriate to represent the multifactorial nature of the pharmacological response. This approach offers a useful framework for supporting clinical decision-making and for strengthening strategies for prevention and early detection of adverse events, laying the groundwork for future research that integrates genetic, clinical, and contextual variables in order to improve the accuracy and clinical utility of the proposed models.

### 5.1. Limitations of the Study

A relevant limitation of the study is related to the availability of clinical information. Comprehensive and sensitive clinical data are generally not found in publicly accessible repositories due to ethical and patient confidentiality considerations. For this reason, the analysis was based on a dataset from a public repository that contains real anonymized information that can be shared without compromising the privacy of individuals.

Consequently, it was not possible to incorporate more in-depth clinical variables, such as detailed medical history, genetic profiles, or longitudinal records, which restricts the approach to the problem to a moderate level of complexity and prevents the application of deep learning architectures that require richer and more structured volumes of information. This limitation conditions the scope of the proposed model and its generalization potential, although it allows establishing a valid initial approach within an ethical and reproducible framework.

A limitation of this study is the absence of external validation of the dataset structure. However, this does not undermine the validity of the analysis, as the data were obtained and harmonized from authoritative and widely used repositories, namely SIDERS and FAERS.

Additionally, the developed models were evaluated only on the available dataset, without having an independent set for external validation. The absence of validation in a different cohort limits the extrapolation of results to other clinical or population settings and restricts the assessment of model robustness in the face of variations in data distribution.

Finally, because the progressive publication of individual patient information is restricted by ethical and confidentiality considerations, the dataset used presents a cross-sectional design. Consequently, it was not possible to carry out a longitudinal follow-up of the clinical status of the patients, which makes it impossible to analyse in detail the temporal evolution of the improvement or possible relapse associated with pharmacological treatment. This limitation restricts the dynamic assessment of side effects and therapeutic response and suggests that future studies should incorporate longitudinal data to deepen the understanding of patterns of clinical progression and occurrence of adverse events.

### 5.2. Public Health Implications

The approach proposed in this study has relevant implications for the field of public health, as it offers a potential tool for the early identification of risks associated with the use of drugs in heterogeneous populations. The probabilistic prediction of side effects makes it possible to anticipate scenarios of greater vulnerability and to guide preventive strategies aimed at specific groups of patients, contributing to the reduction of avoidable adverse events and hospitalizations associated with drug reactions.

Likewise, the incorporation of predictive models in pharmacovigilance systems can strengthen active surveillance and optimize the allocation of health resources by prioritizing interventions in those cases with a higher probability of presenting complications. In this sense, the use of machine learning techniques in population contexts favors the transition from a reactive approach to a preventive model of pharmacological risk management, with a direct impact on improving patient safety and the efficiency of health systems.

## Figures and Tables

**Table 1:** Descriptive analysis for age, dosage, duration of treatment, and improvement score.

	Age	Dosage (mg)	Treatment Duration (Days)	Improvement Score
n.	1000.00	1000.00	1000.00	1000.00
Mean	49.86	352.65	32.38	7.02
Std. Dev.	18.11	295.42	15.70	1.43
Min	18.00	50.00	5.00	2.50
25%	35.00	100.00	19.00	6.10
50%	50.00	250.00	32.00	7.00
75%	66.00	500.00	46.00	8.00
Max	79.00	850.00	59.00	10.00

**Table 2:** Sex distribution and condition of patients.

	Frequency	%	Cumulative %
**Sex**			
Male	523	52.30%	52.30%
Female	477	47.70%	100.00%
**Condition**			
Infection	215	21.50%	21.50%
Pain Relief	208	20.80%	42.30%
Diabetes	207	20.70%	63.00%
Hypertension	194	19.40%	82.40%
Depression	176	17.60%	100.00%

**Table 3:** Chi-squared test for association between qualitative characteristics and the side effects variable.

Variable	*X* ^2^	Dof	*p* Value
Condition ^1^	2533.53	116	*p* < 0.05
Drug	8912.31	406	*p* < 0.05

^1^ Condition refers to the primary clinical indication for which the drug was prescribed.

**Table 4:** Parameters optimized for machine learning models.

Model	Optimal Parameters	Accuracy
Random Forest	model_max_depth: 10	0.345
	model_min_sample_split: 2	
	model_n_estimators: 100	
Decision Tree	model_criterion: gini	0.349
	model_max_depth: None	
	model_min_samples_split: 2	
SVC	model_c: 0.1	0.167
	model_gamma: auto	
	model_kernel: rbf	
KNN	model_metric: Manhattan	0.1325
	model_n_neighbors: 9	
	model_weights: distance	

**Table 5:** Metrics obtained from cross-validation training.

Model	Fold1	Fold2	Fold3	Fold4	Fold5	MA ± SD ^1^	Average Runtime
Random Forest	0.395	0.335	0.32	0.295	0.34	0.337 ± 0.037	~10 s
Decision Tree	0.365	0.33	0.295	0.335	0.33	0.331 ± 0.025	~9 s
SVC	0.17	0.17	0.165	0.165	0.165	0.167 ± 0.002	~20 s
KNN	0.115	0.13	0.1	0.1	0.125	0.114 ± 0.014	~18 s

^1^ MA ± SD: mean accuracy ± standard deviation obtained from five-fold cross-validation.

**Table 6:** Final result of the prediction of machine learning models (Random Forest Output).

Symp. 1	Symp. 2	Symp. 3	Symp. 4	Observed
Nausea (0.75) ^1^	Dizziness (0.18)	Joint pain (0.08)	Swelling (0.00)	Nausea
Tiredness (0.75)	Dizziness (0.15)	Slow heartbeat (0.09)	Fatigue (0.01)	Tiredness
Dry mouth (0.82)	Anxiety (0.10)	Headache (0.07)	Sweating (0.01)	Dry mouth
Low blood sugar (0.74)	Skin rash (0.19)	Nausea (0.08)	Weight gain (0.00)	Low blood sugar
Anxiety (0.81)	Dry mouth (0.1)	Headache (0.09)	Swelling (0.00)	Anxiety

^1^ Symptom (probability).

**Table 7:** Prediction of the most likely symptom by model.

Random Forest		Decision Tree	
Symptom	Probability	Symptom	Probability
Nausea	0.745	Nausea	0.448
Tiredness	0.750	Tiredness	1.000
Dry mouth	0.820	Headache	0.421
Low blood sugar	0.735	Skin rash	0.750
Anxiety	0.805	Anxiety	0.556
**SVC**		**K Nearest-neighbor**	
**Symptom**	**Probability**	**Symptom**	**Probability**
Nausea	0.165	Nausea	0.264
Nausea	0.162	Tiredness	0.271
Nausea	0.152	Dry mouth	0.214
Nausea	0.162	Low blood sugar	0.133
Nausea	0.156	Anxiety	0.242

**Table 8:** Top-k evaluation for adverse effect prediction.

Model	Top 1	Top 3	Top 5
Random Forest	0.345	1.000	1.000
Decision Tree	0.349	1.000	1.000
SVC	0.168	0.406	0.541
KNN	0.133	0.914	0.919

## Data Availability

The dataset used in the present study is publicly available and can be accessed through the following link: https://www.kaggle.com/datasets/palakjain9/1000-drugs-and-side-effects (accessed on 10 March 2026). Likewise, the data can be directly retrieved within the script by using a unique access token, provided that the user has an active Kaggle account.
